# Genome-Wide Analysis of Light-Regulated Alternative Splicing in *Artemisia annua* L.

**DOI:** 10.3389/fpls.2021.733505

**Published:** 2021-09-29

**Authors:** Tingyu Ma, Han Gao, Dong Zhang, Wei Sun, Qinggang Yin, Lan Wu, Tianyuan Zhang, Zhichao Xu, Jianhe Wei, Yanyan Su, Yuhua Shi, Dandan Ding, Ling Yuan, Gangqiang Dong, Liang Leng, Li Xiang, Shilin Chen

**Affiliations:** ^1^Key Laboratory of Beijing for Identification and Safety Evaluation of Chinese Medicine, Institute of Chinese Materia Medica, China Academy of Chinese Medical Sciences, Beijing, China; ^2^Key Lab of Chinese Medicine Resources Conservation, State Administration of Traditional Chinese Medicine of the People’s Republic of China, Institute of Medicinal Plant Development, Chinese Academy of Medical Sciences and Peking Union Medical College, Beijing, China; ^3^School of Life Sciences, Central China Normal University, Wuhan, China; ^4^College of Agriculture, South China Agricultural University, Guangzhou, China; ^5^Guangdong Laboratory for Lingnan Modern Agriculture, Guangzhou, China; ^6^State Key Laboratory of Agricultural Microbiology, Huazhong Agricultural University, Wuhan, China; ^7^Hainan Provincial Key Laboratory of Resources Conservation and Development of Southern Medicine, Hainan Branch of the Institute of Medicinal Plant Development, Chinese Academy of Medical Sciences and Peking Union Medical College, Haikou, China; ^8^Amway (China) Botanical R&D Center, Wuxi, China; ^9^Department of Plant and Soil Sciences, Kentucky Tobacco Research and Development Center, University of Kentucky, Lexington, KY, United States

**Keywords:** *Artemisia annua*, single molecule real-time (SMRT) sequencing, alternative splicing, intron retention, light-regulated, artemisinin

## Abstract

Artemisinin is currently the most effective ingredient in the treatment of malaria, which is thus of great significance to study the genetic regulation of *Artemisia annua*. Alternative splicing (AS) is a regulatory process that increases the complexity of transcriptome and proteome. The most common mechanism of alternative splicing (AS) in plant is intron retention (IR). However, little is known about whether the IR isoforms produced by light play roles in regulating biosynthetic pathways. In this work we would explore how the level of AS in *A. annua* responds to light regulation. We obtained a new dataset of AS by analyzing full-length transcripts using both Illumina- and single molecule real-time (SMRT)-based RNA-seq as well as analyzing AS on various tissues. A total of 5,854 IR isoforms were identified, with IR accounting for the highest proportion (48.48%), affirming that IR is the most common mechanism of AS. We found that the number of up-regulated IR isoforms (1534/1378, blue and red light, respectively) was more than twice that of down-regulated (636/682) after treatment of blue or red light. In the artemisinin biosynthetic pathway, 10 genes produced 16 differentially expressed IR isoforms. This work demonstrated that the differential expression of IR isoforms induced by light has the potential to regulate sesquiterpenoid biosynthesis. This study also provides high accuracy full-length transcripts, which can be a valuable genetic resource for further research of *A. annua*, including areas of development, breeding, and biosynthesis of active compounds.

## Introduction

The main natural source of Artemisinin, a sesquiterpene lactone with peroxide bridge, is the medicinal herb *Artemisia annua* (Miller and Su, 2011; Tu, 2011). Artemisinin is famous for its role in artemisinin combination therapies (ACT), the most effective malaria treatment currently available (Czechowski et al., 2016). Development of new uses of artemisinin and its derivatives, e.g., dihydroartemisinin in the treatment of systemic lupus erythematosus, is still ongoing. These efforts show some potential in anti-tumor, anti-parasitic, anti-fibrosis, and anti-arrhythmic effects (Bin and Hong, 2010). As a result, the demand for artemisinin and its derivatives will increase significantly (Peplow, 2013). Although biosynthesis and chemical synthesis of Artemisinin have seen significant progress in recent years^5,6^, the final steps in the conversion of dihydroartemisinic acid (DHAA) to artemisinin are still non-enzymatic and require extracellular treatments (Graham et al., 2010; Czechowski et al., 2016). For example, *in vitro* biosynthesis of artemisinin is still not possible because yeast cannot provide the special oil oxidization environment required for the synthesis of artemisinin (Paddon et al., 2013; Paddon and Keasling, 2014). Thus, the market supply of artemisinin is currently relied on extraction from *A. annua* (Paddon et al., 2013; Ma et al., 2018; Triemer et al., 2018). Great efforts, particularly in molecular analysis, are needed to understand the biology of *A. annua* in order to improve artemisinin content of the plant and to reduce production cost.

Transcriptional regulation of genes, such as terpenoid synthase, is central to artemisinin biosynthesis (Nagegowda et al., 2008; Nagegowda, 2010; Tan et al., 2015). Thus, research efforts have focused on the *A. annua* transcriptome. Alternative splicing (AS) plays a fundamental role in eukaryote gene regulation (Nilsen and Graveley, 2010; Reddy et al., 2013; Staiger and Brown, 2013). During the process of precursor mRNA maturation, AS produces multiple mature mRNA from the same gene, which then results in the production of different proteins. The alternative splicing of precursor RNA with multiple exons increases the diversity of proteins encoded by organisms and the complexity of regulating gene expression (Brett et al., 2002; Matlin et al., 2005; Keren et al., 2010; Nilsen and Graveley, 2010). In human, nearly all multi-exon genes are alternatively spliced (Pan et al., 2008; Wang et al., 2008), while in plants the corresponding proportion is over 60% (Reddy et al., 2013). AS can occur through different mechanisms, e.g., exon skipping, alternative 3′/5′ splice sites, or intron retention (IR) (Reddy et al., 2013). In contrast to human, in which exon skipping accounts for the largest proportion of alternative splicing products, plants tend to use IR as the main mechanism of AS (Kim and Yin, 2005; Keren et al., 2010; Reddy et al., 2013). For example, the IR rates in Arabidopsis and rice are as high as 64.1 and 55%, respectively (Zhang et al., 2016). AS also participates in the process of antibiotic stress response (Staiger and Brown, 2013; Filichkin et al., 2015). Plants produce secondary metabolites to defend against biological or abiotic stress, e.g., light, plant hormones, temperature, saline and drought stresses (Li et al., 2007; Yu Z. X. et al., 2012; Shen et al., 2016; Vashisth et al., 2018; Lopes et al., 2019). Specifically, the expression of isoforms produced by IR splicing can be regulated depending on source of stress (Filichkin et al., 2010; Abdel-Ghany et al., 2016). For example, light is an important environmental factor that regulates plant physiological adaptation and influences the synthesis of plant secondary metabolites in plant. Light regulation has been demonstrated to promote the accumulation of artemisinin, anthocyanins, ginsenoside and charantin (Cuong et al., 2017; Kochan et al., 2019; Lopes et al., 2019; Sun et al., 2020). Light exposure immediately induced IR, with many alternatively spliced transcripts expressed from genes with functions related to light signaling, suggesting a potential impact on pre-mRNA splicing and photomorphogenic gene regulation in response to light (Petrillo et al., 2014a, b; Godoy Herz et al., 2019).

In recent years high-throughput sequencing technology has proven to be a powerful strategy to study medicinal plant genome, full-length transcriptomes and AS (Wang et al., 2009; Martins et al., 2011; Ozsolak and Milos, 2011; Song et al., 2018; Liu et al., 2021). The main advantage of the most popular high throughput sequencing technology used for transcriptomics, i.e., Next Generation Sequencing, is the ability to study all expressed genes simultaneously. However, current NGS technology suffers from insufficient read length (100–300 bp currently), and the inability to accurately construct full-length splice variants (Pan et al., 2008; Au et al., 2013). The third generation sequencing (TGS) technology, e.g., SMRT sequencing from the PacBio platform (Pacific Biosciences of California, Inc.,^1^), produces a long sequence with much longer read length >8 kb (Au et al., 2010; Mason and Elemento, 2012; Rhoads and Au, 2015). A major drawback of TGS technology, compared with NGS technology, is the high error rate (Au et al., 2010; Koren et al., 2012; Jiao et al., 2013). Two alternatives may be helpful to overcome this problem. First, the length of raw pacbio read is several times longer than the length of most genes; thus, the circular consensus sequence (CCS) mode of pacbio technology could enable a single transcript (cDNA) to be sequenced by multiple passes, and sequencing error of the raw TGS read could be corrected since they were randomly generated (Sharon et al., 2013; Rhoads and Au, 2015). Second, hybrid sequencing, i.e., integrating of NGS sequencing and TGS sequencing technology, could also be applied to the correct the error of PacBio reads (Au et al., 2010, 2013). TGS sequencing technology is widely used in plant AS studies, e.g., *Salvia miltiorrhiza* (Xu et al., 2015), *Zea mays* (Thatcher et al., 2014), *Fragaria ananassa* (Li et al., 2018), *Scutellaria baicalensis* (Gao T. et al., 2019) and *Andrographis paniculata* (Gao H. et al., 2019).

Considering the importance of *Artemisia annua* biology and recent progress in TGS sequencing, we sequenced multiple tissues of *Artemisia annua* to investigate its alternative splicing landscape. Together with current genome assembly and annotation (Shen et al., 2018), in this work we aimed to supply a useful resource to the *Artemisia annua* community with a new genome wide isoform set. Previous studies have shown that different light conditions can affect artemisinin synthesis and accumulation. However, the mechanism by which light regulates artemisinin synthesis is unclear. Many transcription factors have previously been found to regulate artemisinin synthesis at the transcriptional regulatory level, but it is not clear whether AS can affect artemisinin synthesis. Herein, the classification and gene-expression of AS isoforms under different light conditions could provide novel insights in the molecular mechanism of light’s influence on artemisinin synthesis.

## Materials and Methods

### Plant Materials Preparation

The variety of *A. annua* used in this study is a wild type variety originally collected in Hainan (19°56′44″N, 110°13′23″W, 2015) and cultured in Guangxi “Seed cultivation and planting base of ecological of *A. annua*” (25°13′2″N, 109°24′39″W). The seeds were then transplanted to Beijing (40°20′17.8″N, 116°33′40″W) in March 2018 from Guangxi and preserved in the Artemisinin Research Center, China Academy of Chinese Medical Sciences. The seeds were cultured in white light at 25°C for 4 weeks. The light intensity of white light was 50 ± 5 μmol/m^2^s, and the photoperiod was 16/8 h (day/night). Then, the seedlings were transferred to four continuous light treatments for 2 days. After different light treatments, we collected the aboveground parts of 10 seedlings randomly as a sample. The light intensity was 50 ± 5 μmol/m^2^s in all the treatments, including light-emitting diode (LED) red light (wavelength 670 nm), LED blue light (wavelength 470 nm), and LED far-red light (wavelength 735 nm) (Zhang et al., 2017). For the purposes of full-length transcriptome and Illumina short reads analysis, samples of *A. annua* were obtained from four different organs of plant: young leaf, stem, open flower, and root. Each plant organs collected three biological samples for Illumina short reads analysis.

### mRNA-seq Library Preparation and Sequencing

The total RNA from each sample for RNA-seq were extracted from seedlings using RNAprep Pure Plant Kit (Tiangen, Beijing, China) following the manufacturer’s instructions (Zhang et al., 2017). Total RNA in this paper was used for Illumina Hi-seq 2500 platform. We prepared total RNA to construct the sequencing libraries using NEBNext^®^ Ultra^TM^ RNA Library Prep Kit for Illumina^®^ (NEB, MA, United States) with 28S/18S RNA ratio ≥ 1.8, and a minimum integrity number (RIN) value of 8.5 and 250–300 bp insertion element. The integrity of RNA was assessed using the Agilent 2100 Bioanalyzer (Agilent Technologies, CA, United States). Detection of RNA samples, including 1% agarose electrophoresis, was performed to detect degradation and genomic DNA contamination; Nanodrop detects the purity of the RNA (OD260/280 ratio); Qubit quantifies RNA precisely.

In the reaction of synthesizing cDNA, the SMART primers of 3′ terminal oligo (dG) were added beforehand, and reverse transcribed from the RNA3′ terminal with oligo dT as primer. Touching the “hat structure” (methylated G) of eukaryote mRNA at the 5′ end of mRNA adds several (dC) to the end of cDNA. The oligo (dG) of the SMART primer is paired with several C protruding at the end of the synthesized cDNA to form an extension template of the cDNA. The reverse transcriptase automatically converts the template, and the SMART primer continues to extend the cDNA single strand until the end of the primer. In this way, the cDNA contains the starting primer (oligo dT) and SMART primers, which can be amplified by universal primer sequence. The full-length cDNA from pooled poly (A) RNA samples was normalized and subjected to SMRT sequencing using the PacBio RS platform (Novogene, Beijing, China). The full-length cDNA from polymerized (A) RNA samples was normalized and SMRT sequenced by PacBio RS platform (Beijing, China).

### Preprocessing of Single Molecule Real-Time Long Reads

Three steps were conducted to obtain the final SMRT long reads for subsequent analysis. Firstly, circular consensus sequence (CCS) reads were generated from raw subreads using the Pacific Biosciences SMRT-Analysis pipeline version 2.2.0 (Berlin et al., 2015; Ardui et al., 2018). Secondly, full-length reads were identified from the CCS reads by running hmmer_wrapper.py. To define full-length reads, hmmer_wrapper.py requires both 5′ and 3′ primer sequences, as well as a poly (A) tail. The primer sequences and poly (A) tail sequence were then trimmed off prior to further analysis.^2^; finally, PE150 short reads, generated using an Illumina HiSeq 2500, were used to correct full-length reads (PBcR) (Koren et al., 2012). Since this correction step was resource-consuming, we randomly down-sampled illumina short RNA-seq reads to about 12 Gb per tissue for correction. The quality of final full-length reads was assessed by mapping reads to the reference genome using STAR version 2.5.2a (Dobin et al., 2013).

### Isoform Detection and Prediction and Alternative Splicing Type Identification

The SpliceMap-isoform detection and prediction (IDP) pipeline (Au et al., 2013) was used to detect and predict isoforms in four tissues, separately. We initially used SpliceMap to detect the junctions of Illumina short reads obtained from stem, leaf, flower and root samples, and then used IDP to detect and predict isoforms by integrating both Illumina short reads and SMRT long reads. These Illumina RNA-seq reads were all used for SpliceMap analysis, and both full-length reads and corrected full-length reads were merged for IDP. The IDP results indicate the sites where isoforms are located on the genome. We followed our previous work to identify alternative splicing events (Gao H. et al., 2019). Briefly, we used the original annotated genes as reference isoform (hereinafter ref isoform). Original annotation files were downloaded from https://www.ncbi.nlm.nih.gov/genome/?term=Artemisia+annua (Shen et al., 2018). If a gene already has more than one transcript or isoform, we defined the longest isoform as the ref isoform and other isoforms as AS isoforms. We then compared the location information of ref isoforms and AS isoforms to identify the type of AS that might occur in a gene.

### Isoform Quantification and Analysis of Differentially Expressed Isoforms

All ref and AS isoform sequences were combined into a new fasta-format file and quantified using Kallisto version 0.43 (Bray et al., 2016; Jin et al., 2017). A previously published light-treated RNA-seq data were re-calculated with this new *A. annua* isoform set (Zhang et al., 2018). Briefly, plant seedlings were treated with different lights, i.e., FR/far red, R/red, B/blue, WL/white and D/Dark. Each treatment comprised three biological replicates (including 10 seedlings per replicate). In total, 45 RNA-seq libraries were analyzed based on the new *A. annua* isoform set. We also conducted differentially expressed (DE) analysis on FR vs. WL, R vs. WL, B vs. WL as well as WL vs. D, FR vs. D, R vs. D and B vs. D. DE analysis was conducted using R package DEseq2 (Love et al., 2014; Maza, 2016). Genes with adjusted *p*-value being less than 0.05 were kept as differentially expressed ones.

### Analysis of the Intron Retention Isoform Expression Levels of Genes Related to the Artemisinin Biosynthesis Pathway

According to the identified isoforms and their expression, genes involved in artemisinin biosynthesis were manually curated and alternatively spliced isoforms of these genes were selected (Graham et al., 2010; Czechowski et al., 2016, 2018; Ma et al., 2018; Shen et al., 2018). Pheatmap package was used to generate heat maps of isoforms of these genes and the expression patterns of the ref and IR isoforms were analyzed (Kolde, 2015). Enrichment analysis of gene ontology (GO) terms was conducted on differentially expressed IR isoforms using the clusterProfiler package (Yu G. et al., 2012). Functional categories of GO terms with adjusted *p*-value smaller than 0.05 were considered as significantly enriched.

## Results

### Summary of Single Molecule Real-Time Sequencing

To identify AS events in *A. annua*, we applied both Illumina and SMRT RNA-sequencing and analyzed these data in four tissues separately, i.e., leaf, flower, stem and root. We generated ∼15 Gb NGS data and more than 10 Gb TGS data for each tissue (Table 1). For TGS RNA-sequencing data, 7–10 million raw subreads were obtained in each tissue, with median lengths of raw subreads are 1,898 bp, 1,451 bp, 1,093 bp and 1,794 bp in leaf, flower, stem and root, respectively (Table 1). We further generated full length reads from raw subreads (Table 1 and Supplementary Table 1). The number of full-length reads ranges from 341,083 in flower to 412,999 in root, while the median lengths changed to 1,973, 1,956, 2,185, and 1,962 bp in leaf, flower, stem and root, respectively (Table 1 and Supplementary Table 1). We mapped the full length reads to the reference genome and accessed the quality (Supplementary Table 2). Overall, the quality of the reads is high; mapped fractions range from 97.4 to 99.4%, while match rates are around 98 percent. By examining unmapped or deficiently mapped reads manually, we found that a large fraction of these sequences have poly A/G/C/T structure and are therefore difficult to map. We thus corrected these full reads with Illumina short reads. After correction, the number of corrected full-length read ranges from 338,27 in flower to 411,076 in root (Table 1 and Supplementary Table 1). While mapped fraction and mismatch rates only improved by a small proportion, deletion/insertion rate, which may be the result of inaccurate poly-A/G/C/T, decreased (Supplementary Table 2).

**TABLE 1:** Summary of the sequencing libraries statistics of next generation sequencing (NGS) and third generation transcriptome sequencing (TGS) data in leaf, flowers, stem and root in *A. annua.*

**Tissue**	**Illumina read**		**SMRT raw read**			**Full length read**		**Corrected full length read**	
	**Total base**	**Used for correction**	**Num. of reads**	**Total base**	**Median length**	**Num. of reads**	**Median length**	**Num. of reads**	**Median length**
Leaf	∼14G	∼12G	10,171,843	∼20G	1,898	370,515	1,973	369,984	1,963
Flower	∼16G	∼12G	7,532,512	∼12G	1,451	341,083	1,956	338,277	1,923
Stem	∼15G	∼12G	7,075,767	∼10G	1,093	366,742	2,185	352,311	2,013
Root	∼17G	∼12G	7,217,207	∼13G	1,794	412,999	1,962	411,076	1,934

### The New *Artemisia annua* Isoform Annotation Set

The *A. annua* genome was assembled and annotated previously (Shen et al., 2018). This version of annotation has 63,226 genes, of which 3,062 genes comprise more than one isoform (Table 2). However, only 159 AS genes involve intron retention (IR), which contrasts with previous observation showing that ∼30% of all AS events are IR (Keren et al., 2010). Thus, we made use of our newly generated full-length reads to update the current *A. annua* genome annotation and to generate a new *A. annua* isoform annotation set. We identified 13,328 AS events, 11,832 AS isoforms and 7,210 AS genes. Together with the previously published annotation set, here we generated a new *A. annua* annotation set, which consists of 63,226 genes and 75,058 transcripts in total.

**TABLE 2:** Number of AS and IR gene.

	**Num. of AS gene**	**Num. of IR gene**
Leaf	1,613	961
Flower	1,925	1,188
Stem	2,792	2,186
Root	1,940	1,123
All new identified	5,155	3,789
Previously published	3,062	159
Final set	7,210	3,895

**FIGURE 1:**
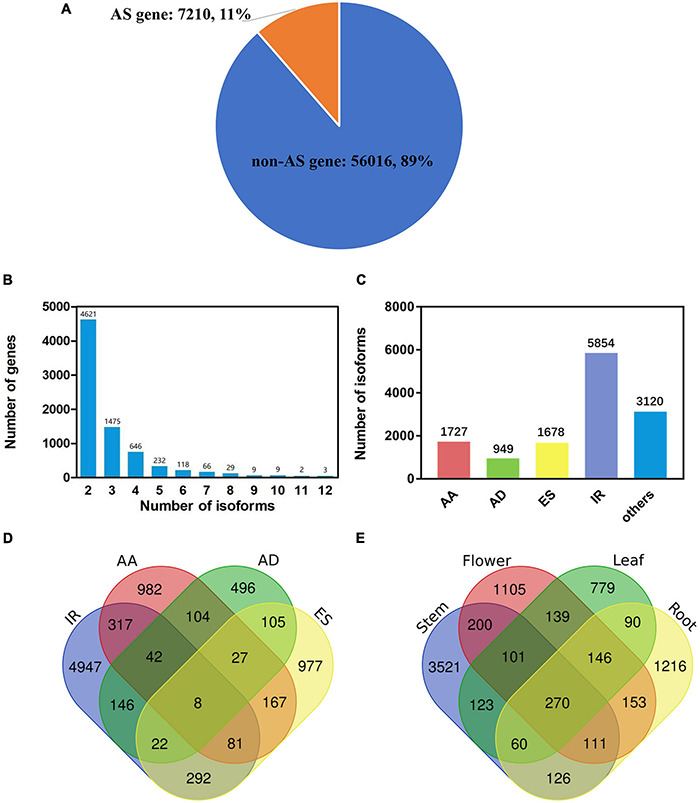
Identification and classification of gene isoforms in *Artemisia annua* L. **(A)** The number and proportion of AS and non-AS gene. **(B)** The number distribution of alternatively spliced isoforms per gene. **(C)** Types of alternatively spliced isoforms: AA, alternative 3′ splice site; AD, alternative 5′ splice site; ES, exon skipping; IR, intron retention. **(D)** The number of isoforms produced by one or more splicing mechanisms. Venn diagram shows the combination of the four types of isoforms. **(E)** The number of new isoforms found in leaf, flower, stem and root. Venn diagram shows the common and specific isoforms in different tissues.

We mined out all possible AS events separately from the four tissues, then combined newly identified events and AS events annotated in the current gene annotation to form a final isoform annotation set (Table 2 and Supplementary Table 3). Our sequencing in the four tissues jointly identified 5,155 genes which comprise at least one AS isoform. Together with the current gene annotation, the new *A. annua* isoform set comprises 7,210 AS genes (Figure 1A and Table 2). Among the 7,210 AS genes, 11,832 AS isoforms were identified (Supplementary Table 3). More than half of all AS genes have only two isoforms, i.e., a reference isoform and a AS isoform. The largest number of isoforms a gene comprises of is 12 (Figure 1B). We further classified all AS events into five types: AA, alternative 3′ splice site; AD, alternative 5′ splice site; ES, exon skipping; IR, intron retention; others. We found a total of 13,328 AS events from 11,832 AS isoforms, while 5,854 AS isoforms involve IR events (Supplementary Tables 3, 4 and Figure 1C). We defined IR genes as genes which bring at least one IR event. Some AS isoforms could consist of multiple AS events or types (Figure 1D); for example, 317 AS isoforms consist of both IR and AA events. Some AS isoforms were independently found in more than one tissue (Figure 1E). For example, 270 AS isoforms were found in all four tissues. The final isoform set consists of 7,210 AS genes, of which 3,895 are IR genes (Table 2).

### Differential Expression of Intron Retention Isoforms Under Light Treatments

**FIGURE 2:**
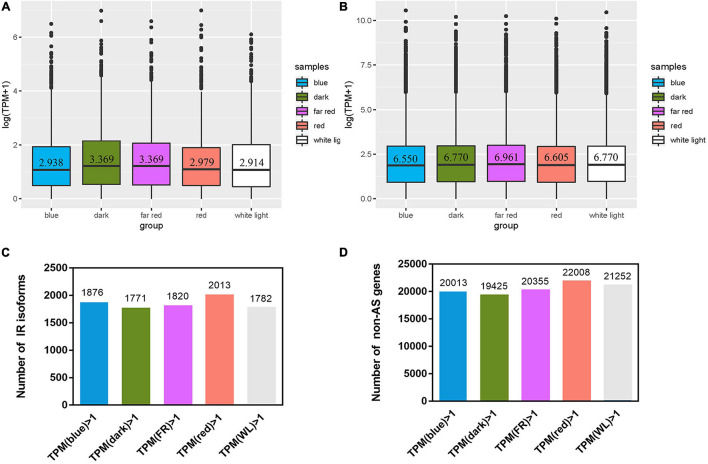
Expression of intron retention (IR) isoforms and non-alternatively spliced (non-AS) genes after light treatment. Isoforms with a TPM > 1 were defined as expressed ones. **(A,B)** Box-plots using log (TPM + 1) as the ordinate show TPM distribution of expressed IR isoforms and non-AS genes under five treatments, respectively. **(C,D)** the number of expressed IR isoforms and non-AS genes, respectively.

In the following part we focused on IR genes and isoform expression under light treatments and of artemisinin synthesis pathway related genes. Five light treatments were conducted, each with three biological replicates (Supplementary Table 4). TPM being 1 was chosen as the cutoff to determine whether a gene is expressed or not. The median TPM of expressed IR isoforms ranges from 2.91 under white light treatment to 3.37 under far-red light treatment (Figure 2A), while that of expressed non-AS genes (which has one isoform per gene) ranges from 6.55 under blue to 6.96 under far red (Figure 2B). The fraction of expressed IR isoforms ranges from 30.2% (1,771) under dark to 34.4% (2,013) under red, while that of non-AS gene ranges from 34.7% (19,425) under dark to 39.3% (22,008) in red (Figures 2C,D and Supplementary Table 5).

**FIGURE 3:**
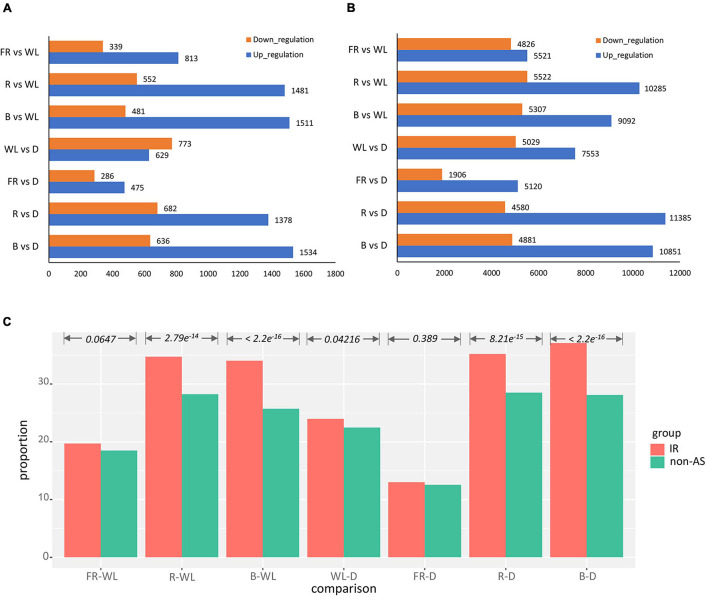
Analysis of differential expression IR isoform expression pattern after light treatment. **(A,B)** Number of up- and down-regulated IR isoforms and non-AS genes after treatment, respectively. **(C)** Proportion of differentially expressed IR isoforms over all IR isoforms vs. the corresponding profortion of non-AS genes. B, blue light; R, red light; FR, far-red light; WL, white light; D, dark.

We further performed differential expression analysis on seven combinations of light treatment: far red vs. white (FR-WL), red vs. white (R-WL), blue vs. white (B-WL), white vs. dark (WL-D), far red vs. dark (FR-D), red vs. dark (R-D), and blue with dark (B-D). In these comparisons, we observed more up-regulated isoforms than down-regulated ones, and this pattern is largely consistent between IR isoforms and non-AS genes apart from WL-D (Figures 3A,B). Moreover, the proportion of IR isoforms which were differentially expressed under different light treatments is higher than that of non-AS genes (Figure 3C and Supplementary Table 6). For instance, 37% of IR isoforms is differentially expressed between the B-D comparison, while the corresponding fraction of non-AS genes is 28% (*p*-value < 2.2e^–16^, Chi-squared test, Figure 3C).

### Gene Ontology Enrichment Analysis of Differentially Expressed Alternative Splicing and Intron Retention Isoforms

Since AS isoforms are more likely to be differentially expressed under light treatment, we used GO enrichment analysis to investigate gene function of differentially expressed AS (Supplementary Table 7) genes, especially IR genes (Table 3 and Supplementary Table 8). For all AS genes, the functional enrichment analysis revealed that transcription regulator activity usually enriches in differentially expressed AS genes since adjusted *p*-values of this GO term (GO: 0140110) are significant in six comparisons, i.e., B-D, B-WL, FR-WL, R-WL and WL-D. Besides, lipid binding (GO: 0008289) is also enriched in four comparisons, i.e., B-D, B-WL, R-D and R-WL. For IR genes, among seven comparisons we found a total of 23 enriched GO terms in six comparisons, belonging to 11 GO annotations (Table 3). No enrichment was found in the FR-D comparison due to the limited number of differentially expressed IR isoforms (Supplementary Table 6). Five GO annotations were enriched in more than one comparison, i.e., nucleotide binding (GO: 0000166) in R-D and R-WL, RNA-binding (GO: 0003723) in R-D and B-WL, lipid metabolic process (GO: 0006629) in B-D, WL-D and B-WL, cellular protein modification (GO: 0006464) in B-D, R-D, B-WL and R-WL, membrane (GO: 0016020) in B-D, R-D, WL-D, B-WL, FR-W and R-WL. These functional enrichments suggest the evolution of IR genes with specific functions in response to light conditions. For instance, we found that three overlapping genes (CTI12_AA107750, CTI12_AA262950 and CTI12_AA419320) regulating sugar transporters have responses to blue, red and far-red light (Supplementary Table 9). The ref isoform of the CTI12_AA107750 gene was down-regulated in blue, red and far-red treatment in contrast to dark treatment (green label). However, the IR isoform of the corresponding gene was up-regulated in blue, red and far red treatment (red label). The ref isoform and IR isoforms of the CTI12_AA262950 gene have a similar regulatory mechanism in blue and red-light treatments in contrast to dark. Similarly, the ref isoform and IR isoforms of the CTI12_AA419320 gene was down-regulated and up-regulated in far-red light treatment compared with dark.

**TABLE 3:** Gene ontology (GO) functional enrichment of differentially expressed IR isoforms.

	**ID**	**Description**	**Gene ratio**	**Bg ratio**	***p*.adjust**
IR_DE_B_D	GO: 0016020	Membrane	204/1,312	4,106/34,885	0.0015
	GO: 0006629	Lipid metabolic process	61/1,312	1,038/34,885	0.0182
	GO: 0009987	Cellular process	152/1,312	3,171/34,885	0.0268
	GO: 0006464	Cellular protein modification process	169/1,312	3,590/34,885	0.0268
	GO: 0005975	Carbohydrate metabolic process	82/1,312	1,565/34,885	0.0273
	GO: 0016787	Hydrolase activity	217/1,312	4,800/34,885	0.0279
IR_DE_R_D	GO: 0016020	Membrane	201/1,277	4,106/34,885	0.0008
	GO: 0006464	Cellular protein modification process	176/1,277	3,590/34,885	0.0015
	GO: 0003723	RNA binding	47/1,277	766/34,885	0.0123
	GO: 0016301	Kinase activity	136/1,277	2,832/34,885	0.0141
	GO: 0000166	Nucleotide binding	263/1,277	6,143/34,885	0.043
	GO: 0005623	Cell	22/1,277	315/34,885	0.043
IR_DE_WL_D	GO: 0016020	Membrane	142/896	4,106/34,885	0.0113
	GO: 0006629	Lipid metabolic process	44/896	1,038/34,885	0.0378
IR_DE_B_WL	GO: 0016020	Membrane	201/1,241	4,106/34,885	0.0001
	GO: 0006629	Lipid metabolic process	59/1,241	1,038/34,885	0.0132
	GO: 0006464	Cellular protein modification process	164/1,241	3,590/34,885	0.014
	GO: 0003723	RNA binding	44/1,241	766/34,885	0.0257
	GO: 0006810	Transport	141/1,241	3,102/34,885	0.0257
IR_DE_FR_WL	GO: 0016020	Membrane	120/729	4,106/34,885	0.0071
IR_DE_R_WL	GO: 0006464	Cellular protein modification process	170/1,272	3,590/34,885	0.019
	GO: 0016020	Membrane	188/1,272	4,106/34,885	0.0237
	GO: 0000166	Nucleotide binding	266/1,272	6,143/34,885	0.0313

*Gene Ratio, The ratio of the number of differentially expressed genes associated with this term to the total number of differentially expressed genes; Bg Ratio, The ratio of the number of background genes associated with this term to the total number of all background genes; *p*.adjust, adjust *p*-values for multiple comparisons.*

### Analysis of Intron Retention Genes Related to Artemisinin Synthesis Pathway

**FIGURE 4:**
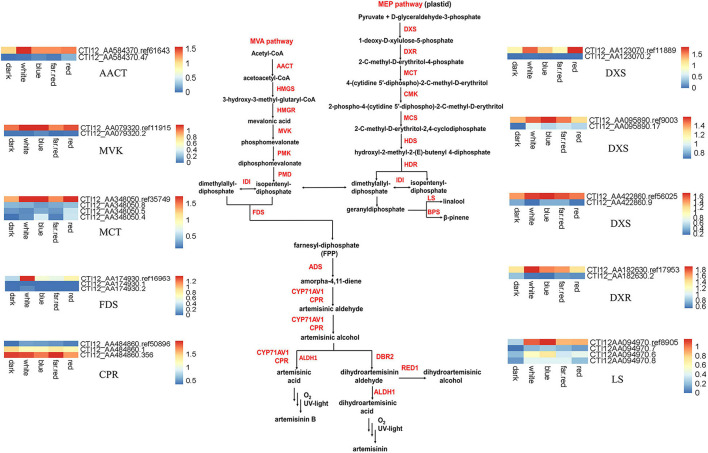
Expression heatmap of the isoforms involved in artemisinin synthesis. The suffix “ref” indicates the reference isoform. For every gene, the reference isoform present in genome annotation is signed with the suffix “ref” or “rna” plus a series of number, while other isoforms newly identified are signed with pure number in its suffix. For example, CTI12_AA584370.ref61643 is the reference isoform of AACT while CTI12_AA584370.47 is a newly identified AS isoform. AACT, Acetyl-CoA C-acetyltransferase; HMGS, 3-Hydroxy-3-methylglutaryl-CoA synthase; HMGR, 3-Hydroxy-3-methylglutaryl-CoA reductase; MK, MVA kinase; PMK, Phospho-MVA kinase; MDC, Diphospho-MVA decarboxylase; DXS, 1-Deoxy-D-xylulose-5-phosphate synthase; DXR, 1-Deoxy-D-xylulose 5-phosphate reductoisomerase; MCT, 2-C-methyl-D-erythritol 4-phosphate cytidylyltransferase; CMK, 4-(Cytidine-5-diphospho)-2-C-methyl-D-erythritol kinase; MDS, 2-C-methyl-D-erythritol-2,4-cyclodiphosphate synthase; HDS, 4-Hydroxy-3-methylbut-2-enyl-diphosphate synthase; HDR, 4-Hydroxy-3-methylbut-2-enyl diphosphate reductase; GGPPS (E, E, E)-geranylgeranyl diphosphate synthase.

We compared the AS genes we identified with genes predicted to be involved in the biosynthesis pathway for the general sesquiterpenes precursor FPP and artemisinin synthesis. Among 24 genes related to artemisinin synthesis pathway and its upstream MVA and MEP pathways (Supplementary Figure 3), fifteen genes with AS behaviors were identified. Nine AS genes in the MEP pathway were identified, belonging to the 1-Deoxy-D-xylulose 5-phosphate synthase (DXS), 1-deoxy-D-xylulose- 5-phosphate reductoisomerase (DXR), 4-(Cytidine 5-diphospho)-2-C-methyl-D-erythritol kinase (CMK), 2-C-methyl-D-erythritol-4-(cytidyl-5-diphosphate) transferase (MCT), 2-C-methyl-D-erythritol-2,4-cyclodiphosphate synthase (MCS), and R-linalool synthase (LS) gene families (Supplementary Table 10). Five genes in the MVA pathway were identified, belonging to the acetoacetyl-CoA thiolase (AACT), mevalonate kinase (MVK), and farnesyl diphosphate synthase (FDS) gene families (Supplementary Tables 10, 11). One gene in the artemisinin synthesis pathway, belonging to the cytochrome P450 reductase (CPR) gene family, was identified. These genes produced a total of 39 isoforms, including 16 IR isoforms. The CPR gene was most highly expressed is an AS isoform, and the remaining genes have highest expression of the ref isoform (Figure 4).

Ten genes were identified that produce IR type isoforms in the artemisinin pathway (Figure 4). The expression of ref isoforms produced by CTI12_AA095890 (DXS), CTI12_AA182630 (DXS), CTI12_AA123070 (DXS), CTI12_AA422860 (DXR), CTI12_AA094970 (LS), CTI12_AA348050 (MCT), CTI12_AA584370 (AACT), CTI12_AA079320 (MVK), CTI12_AA174930 (FDS), and CTI12_AA484860 (CPR) genes, were up-regulated (red label), compared with dark treatment (Supplementary Table 10). Several IR isoforms were alternatively expressed under different light treatments. For example, under white light treatment, expression of the IR isoforms produced by CTI12_AA095890 (DXS) and CTI12_AA094970.6 (LS) genes were up-regulated, while the expression of the IR isoforms produced by CTI12_AA422860 (DXS), CTI12_AA182630 (DXR) and CTI12_AA484860 (CPR) genes were down-regulated. In addition, more IR isoforms are up-regulated in blue light, such as CTI12_AA348050 (MCT), CTI12_AA463490 (MVK), CTI12_AA422860 (DXS), CTI12_AA484860 (CPR) and CTI12_AA123070 (LS), compared with white light (Supplementary Table 10).

Interestingly, the ref isoform of the CPR gene was up-regulated and the IR isoform down-regulated in all light treatments when compared with dark (Supplementary Tables 10, 11). However, the expression of IR isoform produced by CPR gene was up-regulated when contrasted with white light (Supplementary Table 10). CPR has been hypothesized to serve as a redox partner for CYP71AV1, which catalyzes the conversion of amorphadiene to more oxygenated products in *A. annua* (Paddon et al., 2013). Moreover, if a gene has multiple isoforms, i.e., more than one IR isoform, these IR isoforms may contribute in the same or opposite ways under different light conditions as in the CPR or LS cases (Supplementary Table 10). In the artemisinin pathway, we observed genes that produced IR isoforms, e.g., nine genes in the MEP pathway, five genes in the MEP pathway and one gene in the artemisinin synthesis pathway. More IR isoforms are produced in the upstream biosynthesis pathway of artemisinin. Compared with dark, we found the IR isoforms of all 10 genes are up-regulated under light treatment. More IR isoforms were up-regulated under blue and red-light treatments. However, only blue light treatment has more IR expression up-regulated when we compared with white light. One main consequence of IR is the occurrence of premature stop codon (PTC) in retained intron (Supplementary Figures 1, 2). We checked whether PTC exists in IR genes related to the artemisinin synthesis pathway. Seven genes were found to bear PTC in their IR isoforms. For instance, three IR isoforms of the LS (CTI12_AA094970) gene, i.e., CTI12_AA094970.6, CTI12_AA094970.7, and CTI12_AA094970.8, retain the second intron, leading to the PTC of these AS isoforms. Thus, all these three isoforms may produce a truncated protein which lacks the 3rd to 6th exons. PTC-present isoforms may also produce pre-mRNAs which will further subject to non-sense-mediated mRNA decay (NMD) and the expression of this gene thus could be appropriately regulated by these PTC-present isoforms induced decay (Isken and Maquat, 2008). These results imply that IR are involved in expression regulatory mechanism to control the metabolism of sesquiterpenes.

## Discussion

The accumulation of artemisinin is unique in *A. annua*, and the yield of artemisinin largely determines the medicinal value of *A. annua* (Graham et al., 2010; Czechowski et al., 2016, 2018). Since light has a positive effect on the accumulation of secondary metabolites (Li et al., 2017; Yoo et al., 2019; Zhang et al., 2019), the study of the molecular mechanism of the effect of light on artemisinin accumulation is of great significance for basic science and practical applications. In this study, we used both NGS and SMRT-based RNA-seq to identify transcripts of *A. annua* under different light conditions including blue, red, far-red, white light and dark treatments (Zhang et al., 2017), and analyzed the overall transcriptional level of each gene. By combining Illumina and SMRT platforms for transcriptome sequencing of leaf, flower, stem, root in *A. annua*, we generated a new isoform set. Our study identified 11,832 AS events in total, of which 5,854 were IR isoforms (Figure 1 and Table 2). File of a total of 11,832 AS isoforms sequences is stored in Supplementary_AS_sequence.fasta. We found that IR isoforms accounted for 48.48% of all AS isoforms, representing the most frequent AS event is IR in *A. annua*. This result is consistent with previous findings in other plants (Filichkin et al., 2010; Chang et al., 2014; Gao H. et al., 2019). We found that IR isoform expression tends to be differentially expressed under light treatment. Comparing with the current annotation that records only 159 IR genes (Table 2), our new AS dataset is more reliable based on our knowledge of plant AS patterns and could sufficiently improve the *A. annua* genome annotation.

Former studies have shown that environmental stress can affect AS global pattern (Palusa et al., 2007; Filichkin et al., 2010, 2015; Duque, 2011; Reddy et al., 2013; Staiger and Brown, 2013; Ding et al., 2014). Light not only provides energy for plants, but also is a key environmental factor (Filichkin et al., 2010; Chang et al., 2014; Dietz, 2015; Gao H. et al., 2019). There are reported that the deletion of SAD1 which is a key gene involved in ABA signal pathway results in a genome-wide increase of AS (Cui et al., 2014). Our work also revealed that light possibly affect the production of AS particularly IR. Comparing with the non-AS isoforms, there is a greater proportion of AS gene expression regulated by light, which suggests that light can influence not only the production of AS but also its gene expression (Figure 3C). This finding could broaden our insight into how light regulates the behavior of plants. Ten genes were identified that produce IR type isoforms in the artemisinin pathway, but only one is in the artemisinin downstream pathway. This may be due to the upstream is the common pathway of many metabolites and can be affected by more environmental regulatory factors, so as to quick respond to the changes; while the downstream specific artemisinin synthesis pathway may not require too many regulatory changes. Moreover, we also found that the IR genes in the artemisinin pathway was basically high in the expression of reference isoform, while the expression of AS isoform was generally lower. The expression of AS reference and AS isoforms in the artemisinin pathway were higher in most light than in the dark, especially in red and blue light, which was consistent with the most obvious effect of blue and red light on the accumulation of artemisinin. This indicates that the regulation effect of AS on different transcripts is the same on the synthesis pathway of light, which is more conducive to the additive effect on the synthesis regulation of artemisinin and the rapid regulation of light on artemisinin.

Plants have distinct sets of photoreceptors for several parts of the light spectrum, ranging from near-UVB (280–315 nm) to far-red (FR) (∼750 nm) wavelengths. Plants mainly use red and blue light for photosynthesis. At the same time, red and blue light, as a signal, play an important role in many biological processes such as seed germination, de-etiolation, phototropism and flowering (Wit et al., 2016). In Figure 3A, red and blue light can induce more IR isoforms up-regulated than other light treatments, indicating that the transduction of red and blue light signals to plants may depend on the AS. Also, in Figure 3C, red and blue light treatments can induce more differentially expressed IR isoforms. Red and blue light can induce more physiological responses in plants and cause more changes in the expression of AS. Besides, our analysis show that lipid related functions such as lipid binding or lipid metabolic process are enriched in differentially expressed AS or IR genes (Supplementary Tables 7, 8). Lipids, which are major and essential constituents of all plant cells, not only provide structural integrity and energy for various metabolic processes but can also function as signal transduction mediators (Bigay and Antonny, 2012). There is a lot of lipid synthesis and metabolism in chloroplasts. Chloroplast is the site of photosynthesis in plants, and its growth and development are also regulated by light (Chen et al., 2018). Therefore, light changes can cause many lipids synthesis and metabolism gene expression changes. Also, PA, one of the central molecules in lipid biosynthesis, not only facilitates transport of lipids across membranes (Awai et al., 2006) but also plays an important role in biotic and abiotic stress responses. Light is also a stress to plants, especially ultraviolet light. It is possible that light also mediates stress responses in plants through lipid signaling, and therefore, light may affect the expression of many genes related to lipid metabolism. Light is one of the important factors that regulate plant growth and development. Light exposure immediately induced alternative splicing, with many alternatively spliced transcripts expressed from genes with functions related to light signaling, suggesting a potential impact on pre-mRNA splicing and photomorphogenic gene regulation in response to light (Yoshimura et al., 2011; Petrillo et al., 2014a, b; Godoy Herz et al., 2019). Light promotes RNA polymerase II (Pol II) elongation in the affected genes, whereas in darkness, elongation is lower (Godoy Herz et al., 2019). Therefore, Photoreceptors strictly regulate gene expression to control light morphological response. These results jointly suggest that AS may play an important role in the transduction of light signals.

Overall, the number of up-regulated IR isoforms was more than the total number of down-regulated IR isoforms after blue, red, and far-red light treatments. GO enrichment indicated that some genes with special functions can produce multiple isoforms. In the artemisinin biosynthetic pathway, a total of 10 genes underwent AS to produce 26 isoforms, including 16 IR isoforms, two AA isoforms, and eight other isoforms (Supplementary Tables 10, 11). Our research suggests that the expression of IR isoforms is regulated by blue, red, and far-red light, which may also be involved in the biosynthesis of artemisinin and other plant secondary metabolite synthesis (Chen et al., 2013; Liu et al., 2018). For example, genes such as AACT and DXS in the MVA and MEP pathways exhibited IR isoforms (Figure 4). A recent study reported that most of the IR isoforms in Arabidopsis are predicted to be targets of NMD to regulate mRNA stability as they are subjected to NMD to eliminate incomplete transcripts (Filichkin et al., 2010; Syed et al., 2012; Ge and Porse, 2014; Zhu et al., 2020). These eliminated IR isoforms may contain functional domains important for artemisinin biosynthesis by influencing specific steps of the pathway.

The analysis of AS in *A. annua* has generated valuable insights regarding plant transcriptomes, and these findings provide a foundation for further analyses. In future work, functional verification of IR isoforms will be conducted in the artemisinin pathway and regulatory genes. In conclusion, this study provides not only new insights into the regulation of AS in artemisinin biosynthesis, but also valuable genetic resources for further exploration of functional genomics in *A. annua*.

## Data Availability Statement

The raw sequencing datasets of *A. annua* reported in this paper have been submitted to the Sequence Read Archive from the NCBI under the accession number PRJNA752933.

## Author Contributions

SC, LX, and LL conceived the study. TM, HG, DZ, DD, LW, YYS, and GD performed the sample preparation of NGS and SMRT sequencing. HG, TM, DZ, LL, and TZ analyzed the data. WS, QY, LY, JW, ZX, and YHS contributed to discussion and language polishing. TM, HG, DZ, and LL wrote the manuscript. All authors contributed to the article and approved the submitted version.

## Conflict of Interest

YYS and GD were employed by the Amway (China) Botanical R&D Center. The remaining authors declare that the research was conducted in the absence of any commercial or financial relationships that could be construed as a potential conflict of interest.

## Publisher’s Note

All claims expressed in this article are solely those of the authors and do not necessarily represent those of their affiliated organizations, or those of the publisher, the editors and the reviewers. Any product that may be evaluated in this article, or claim that may be made by its manufacturer, is not guaranteed or endorsed by the publisher.

## Footnotes

1
http://www.pacificbiosciences.com/

2
https://github.com/Magdoll/PacBio-generic/blob/master/hmmer_wrapper.py

## Supplementary Material

Supplementary Figure 1 Sequence alignment of PTC-present isforms and their corresponding reference isoforms. Location of PTC is highlighted in red rectangle.

Supplementary Figure 2 Gene structure of PTC-present isforms and their corresponding reference isoforms. Location of PTC is indicated by black arrow.

Supplementary Figure 3 Expression heatmap of genes related to MVA, MEP and artemisinin synthesis pathways.

Supplementary Table 1 Pre-processed of full-length reads and corrected full-length reads data in leaf, flowers, stem and root of *A. annua*.

Supplementary Table 2 Summary of Illumina RNA-seq and single-molecule real-time (SMRT) full-length read and corrected full-length reads quality mapping to the reference genome as determined by STAR.

Supplementary Table 3 The alternative splicing isoforms and splicing types identified by Isoform Detection and Prediction (IDP).

Supplementary Table 4 The expression level of a total of 11,832 alternative splicing isoforms involved in the *A. annua.*

Supplementary Table 5 Genome-wide quantitative analysis of alternative splicing isoforms produced by different light treatments. The table shows that blue, red, far red, white light and dark were used to treat *A. annua* seedlings for 48 h. There were three biological duplicates for each treatment. Kallisto was used to quantify 75,058 transcripts, and the mean value of TPM was taken as the expression of transcripts. Set TPM = 1 to the cut-off value, and the isoform (alternative splicing) of TPM > 1 is considered to be expressed.

Supplementary Table 6 The total number of up-regulated and down-regulated IR isoforms and non-AS genes after treatment with blue, red, far-red, white light and dark. The table shows the total number of up- and down-regulated IR isoforms and non-AS genes after different light treatments, which were used to Supplementary Figure 3.

Supplementary Table 7 Functional enrichment of differentially expressed AS genes. The table shows gene enrichment results corresponding to all differentially expressed AS isoforms. Gene Ratio: The ratio of the number of differentially expressed genes associated with this term to the total number of differentially expressed genes; which had GO annotations; Bg Ratio: The ratio of the number of background genes associated with this term to the total number of all background genes which had GO annotations; *p*.adjust: adjust *p*-values for multiple comparisons.

Supplementary Table 8 Alternative splicing isoforms of up-regulated and down-regulated genes in Gene ontology (GO) functional enrichment results. The table shows gene enrichment results corresponding to all differentially expressed IR isoforms were either up-regulated or down-regulated after blue, red, far-red, white light and dark treatment.

Supplementary Table 9 The overlapping genes in Gene ontology (GO) enrichment analysis result.

Supplementary Table 10 The alternative splicing type and expression level of the genes involved in the *A. annua* (dark as a control).

Supplementary Table 11 The alternative splicing type and expression level of the genes involved in the *A. annua* (white as a control).

Supplementary_AS_sequence.fasta Fasta-format sequence file of a total of 11,832 alternative splicing isoforms involved in the *A. annua.*
