# Supplementary figures and images for: Genome-Wide Analysis of Light-Regulated Alternative Splicing in Artemisia annua L
Source: Front Plant Sci. 2021 Sep 29;12:733505. doi: 10.3389/fpls.2021.733505 (PMC8511310; doi:10.3389/fpls.2021.733505)

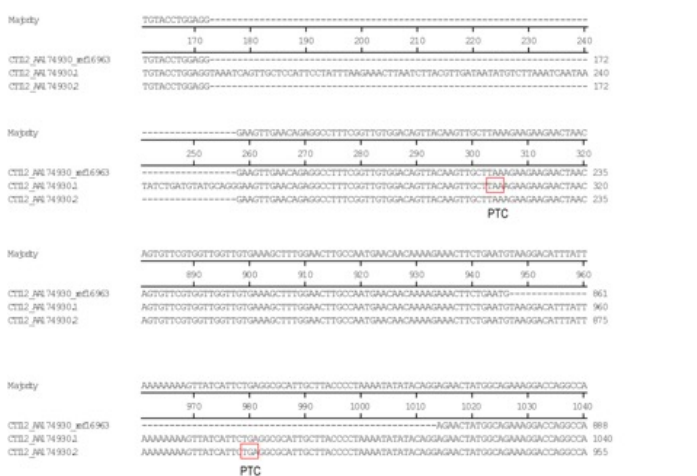

## CTI12\_AA484860

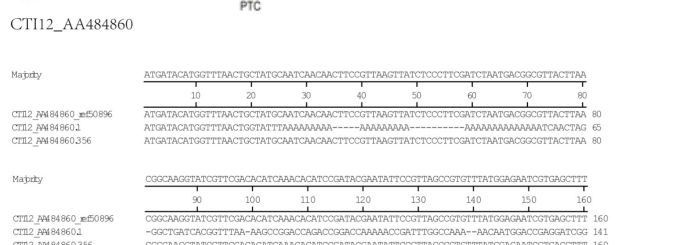

## CTD2 AM84860 ref50896

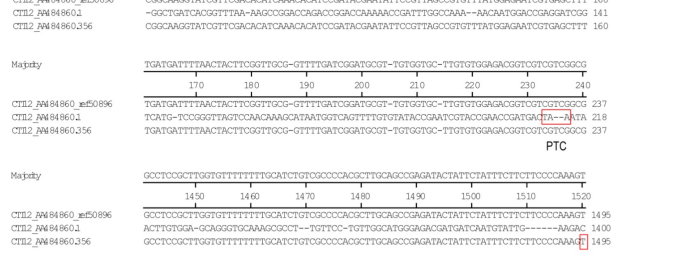

## Majority

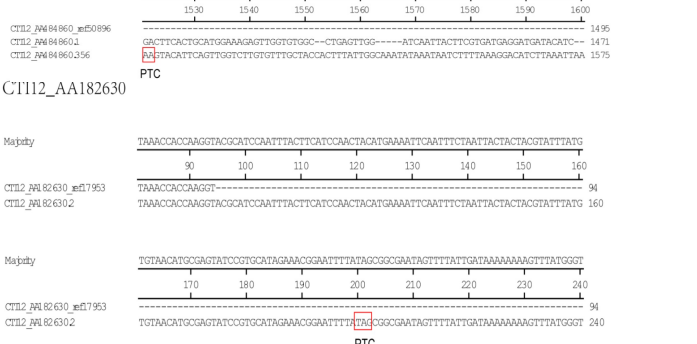

Supplement: Supplementary Figure 1 — Sequence alignment of PTC-present isforms and their corresponding reference isoforms. Location of PTC is highlighted in red rectangle. [file Data_Sheet_1.PDF]

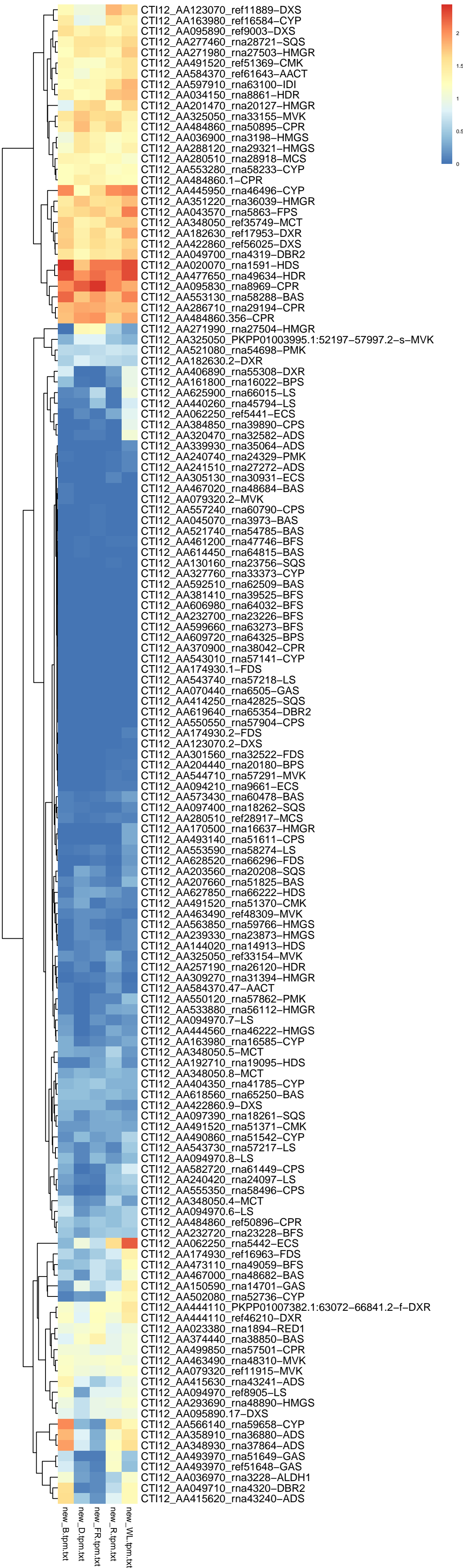

Supplement: Supplementary Figure 3 — Expression heatmap of genes related to MVA, MEP and artemisinin synthesis pathways. [file Data_Sheet_3.PDF]
