# Supplementary material for: Genome-Wide Analysis of Light-Regulated Alternative Splicing in Artemisia annua L
Source: Front Plant Sci. 2021 Sep 29;12:733505. doi: 10.3389/fpls.2021.733505 (PMC8511310; doi:10.3389/fpls.2021.733505)
Supplement: Supplementary Figure 2 — Gene structure of PTC-present isforms and their corresponding reference isoforms. Location of PTC is indicated by black arrow. [file Data_Sheet_2.PDF]

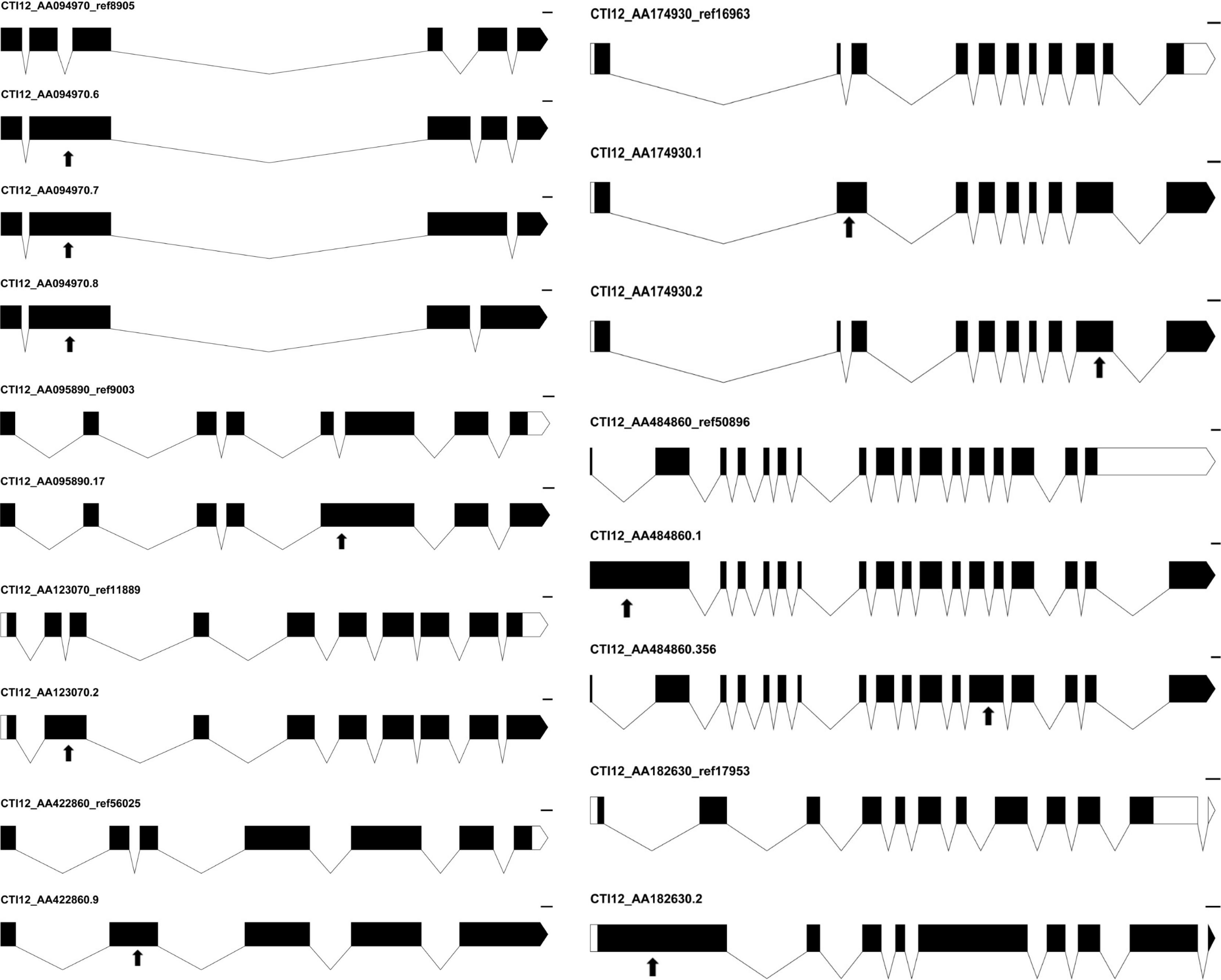
CTI12_AA094970_ref8905
CTI12_AA094970.6
CTI12_AA094970.7
CTI12_AA094970.8
CTI12_AA095890_ref9003
CTI12_AA095890.17
CTI12_AA123070_ref11889
CTI12_AA123070.2
CTI12_AA422860_ref56025
CTI12_AA422860.9
CTI12_AA174930_ref16963
CTI12_AA174930.1
CTI12_AA174930.2
CTI12_AA484860_ref50896
CTI12_AA484860.1
CTI12_AA484860.356
CTI12_AA182630_ref17953
CTI12_AA182630.2
